# Barriers and facilitators to implementing shared decision-making in clinical practice: a systematic review of health professionals' perceptions

**DOI:** 10.1186/1748-5908-1-16

**Published:** 2006-08-09

**Authors:** Karine Gravel, France Légaré, Ian D Graham

**Affiliations:** 1 Research Centre of the Centre Hospitalier Universitaire de Québec, Québec, Canada; 2 Department of Family Medicine, Université Laval, Québec, Canada; 3 Faculty of Health Sciences, University of Ottawa, Ottawa, Canada

## Abstract

**Background:**

Shared decision-making is advocated because of its potential to improve the quality of the decision-making process for patients and ultimately, patient outcomes. However, current evidence suggests that shared decision-making has not yet been widely adopted by health professionals. Therefore, a systematic review was performed on the barriers and facilitators to implementing shared decision-making in clinical practice as perceived by health professionals.

**Methods:**

Covering the period from 1990 to March 2006, PubMed, Embase, CINHAL, PsycINFO, and Dissertation Abstracts were searched for studies in English or French. The references from included studies also were consulted. Studies were included if they reported on health professionals' perceived barriers and facilitators to implementing shared decision-making in their practices. Shared decision-making was defined as a joint process of decision making between health professionals and patients, or as decision support interventions including decision aids, or as the active participation of patients in decision making. No study design was excluded. Quality of the studies included was assessed independently by two of the authors. Using a pre-established taxonomy of barriers and facilitators to implementing clinical practice guidelines in practice, content analysis was performed.

**Results:**

Thirty-one publications covering 28 unique studies were included. Eleven studies were from the UK, eight from the USA, four from Canada, two from the Netherlands, and one from each of the following countries: France, Mexico, and Australia. Most of the studies used qualitative methods exclusively (18/28). Overall, the vast majority of participants (n = 2784) were physicians (89%). The three most often reported barriers were: time constraints (18/28), lack of applicability due to patient characteristics (12/28), and lack of applicability due to the clinical situation (12/28). The three most often reported facilitators were: provider motivation (15/28), positive impact on the clinical process (11/28), and positive impact on patient outcomes (10/28).

**Conclusion:**

This systematic review reveals that interventions to foster implementation of shared decision-making in clinical practice will need to address a broad range of factors. It also reveals that on this subject there is very little known about any health professionals others than physicians. Future studies about implementation of shared decision-making should target a more diverse group of health professionals.

## Background

Shared decision-making (SDM) is defined as a decision making process jointly shared by patients and their health care providers[1]. It aims at helping patients play an active role in decisions concerning their health[2], which is the ultimate goal of patient-centered care[3]. Shared decision-making rests on the best evidence of the risks and benefits of all the available options[4]. It includes the following components: establishing a context in which patients' views about treatment options are valued and deemed necessary, transferring technical information, making sure patients understand this information, helping patients base their preference on the best evidence; eliciting patients' preferences, sharing treatment recommendations, and making explicit the component of uncertainty in the clinical decision-making process[5]. A Cochrane systematic review of 34 randomized controlled trials of shared decision-making programs (also known as decision aids) indicates that compared to usual care or simple information leaflets, these programs: 1) improved knowledge, 2) produced more realistic expectations, 3) lowered decisional conflict, 4) increased the proportion of people active in decision-making, 5) reduced the proportion of people who remained undecided, and 6) produced greater agreement between values and choice[6].

Population-based and clinically-based surveys have shown that a significant proportion of respondents would like to play an active role in decisions concerning their health [7-9]. Although the nature of the problem may influence the amount of control patients want in making decisions for themselves[10,11], more and more individuals recognize that they are the best judges of their values when deliberating over a health care decision[12,13]. Indeed, as Deber (1996) pointed out, making decisions about one's own health consists of "problem-solving" and "decision making that requires the contribution of patients' values and preferences"[14]. While most patients do not wish to be involved in "problem-solving," most would like to be involved in the decision-making process[14]. In a recently published review on optimal matches of patient preferences for information, decision making, and interpersonal behaviour[15], findings from 14 studies showed that a substantial group of patients (26% to 95% with a median of 52%) was dissatisfied with the information given (in all aspects) and reported a desire for more information. In the same review, findings from six studies showed that the better the match between the information that was desired and the information that was received, the better the patient outcomes[15].

Nonetheless, shared decision-making has not yet been widely adopted by health care professionals[10,16-21]. If shared decision-making is desirable, more will need to be done to understand what factors hinder or facilitate its implementation in clinical practices[22]. Therefore, we sought to systematically review studies that reported on health professionals' perceived barriers and facilitators to implementing shared decision-making in their clinical practice.

## Methods

### Search strategy

Covering the period from 1990 to March 2006 and based on a list of 51 key articles in the field of shared decision-making (including a list of 17 studies that dealt with barriers and/or facilitators to implementing shared decision-making in clinical practice), specific search strategies were developed by an information specialist for the following databases: PubMed, Embase, CINHAL, et PsycINFO (see Additional file 1). The information specialist estimated that the proportion of retrieved articles that met our minimum definition of a key article in the field of shared decision-making (positive predictive value) was about 10%–20%, depending on the database. For Pubmed, the sensitivity of search strategy was 100% (proportion of pre-identified key articles in the field of shared decision-making that were identified by his search strategy). In other words, all 51 articles provided to the information specialist were captured by his search strategy. Using the free text words "shared decision-making" or "participation of patient in decision" or "decision aids" or "decision support," Dissertation Abstracts also were searched. References from included studies and review articles[22,23] were scanned.

### Selection criteria

A study was eligible for inclusion in the review if: 1) it was an original collection of data, 2) participants included health professionals, and 3) results included perceived barriers and/or facilitators to shared decision-making. Shared decision-making was defined in an inclusive manner as a joint process between health professionals and patients to make decisions[5,24,25], or as decision support interventions such as decision aids[6], or as the active participation of patients in decision making. We did not restrict our search and inclusion of studies to those reporting as their main objective the assessment of barriers and facilitators to shared decision-making. Thus, we included studies that provided usable data for either of these two outcomes. No study design was excluded, and only studies in French and English were assessed. When more than one publication described a single study and each presented the same data, we included only the most recent publication. However, when more than one publication described a single study but each presented new and complementary data, we included them all.

### Study identification and data extraction

One individual (KG) screened all references. Two reviewers (FL and KG) extracted data independently using a data extraction sheet. At the time this review was conducted and to the best of our knowledge, there was no taxonomy for assessing barriers and facilitators to the implementation of shared decision-making in clinical practice. Therefore, a data extraction sheet was created by using a template analytic approach, "beginning with a basic set of codes based on *a priori *theoretical understanding and expanding on these codes by readings of the text"[26]. The beginning set of *a priori *codes was based on a taxonomy of barriers and facilitators to implementing clinical practice guidelines in actual practice[27,28]. This taxonomy had been used successfully to study factors affecting general practitioners' decisions about plain radiography for back pain by Espeland and colleagues (2003), who concluded that it compared well to other taxonomies[28]. Following previous work by one of the authors[29], we further enriched this taxonomy with some of the attributes of innovations (Table 1)[30].

**Table 1:** Taxonomy of barriers and facilitators and their definitions

**Knowledge**	
Lack of awareness	Inability to correctly acknowledge the existence of shared decision-making (SDM) [27]
Lack of familiarity	Inability to correctly answer questions about SDM content, as well as self-reported lack of familiarity [27]
Forgetting	Inadvertently omitting to implement SDM [41]
**Attitudes**	
Lack of agreement with specific components of shared decision-making	
• Interpretation of evidence	Not believing that specific elements of SDM are supported by scientific evidence [27]
• Lack of applicability	
○ Characteristics of the patient	Lack of agreement with the applicability of SDM to practice population based on the characteristics of the patient [27]
○ Clinical situation	Lack of agreement with the applicability of SDM to practice population based on the clinical situation [27]
• Asking patient about his/her the preferred role in decision-making	Lack of agreement with a specific component of SDM such as asking patients about their preferred role in decision-making [27]
• Asking patient about support or undue pressure	Lack of agreement with a specific component of SDM such as asking patients about support and/or undue pressure [27]
• Asking about values/clarifying values	Lack of agreement with a specific component of SDM such as asking patients about values [27]
• Not cost-beneficial	Perception that there will be increased costs if SDM is implemented [28]
• Lack of confidence in the developers	Lack of confidence in the individuals who are responsible for developing or presenting SDM [27]
Lack of agreement in general	
• "Too cookbook" – too rigid to be applicable	Lack of agreement with SDM because it is too artificial [27]
• Challenge to autonomy	Lack of agreement with SDM because it is a threat to professional autonomy [27]
• Biased synthesis	Perception that the authors were biased [27]
• Not practical	Lack of agreement with SDM because it is unclear or impractical to follow [28]
• Total lack of agreement with using the model (not specified why)	Lack of agreement with SDM in general (unspecified) [27]
Lack of expectancy	
• Patient's outcome	Perception that performance following the use of SDM will not lead to improved patient outcome [27]
• Health care process	Perception that performance following the use of SDM will not lead to improved health care process [28]
• Feeling expectancy	Perception that performance following the use of SDM will provoke difficult feelings and/or does not take into account existing feelings [28]
Lack of self-efficacy	Belief that one cannot perform SDM [27]
Lack of motivation	Lack of motivation to use SDM or to change one's habits [27]
**Behaviour**	
External barriers	
• Factors associated with patient	
○ Preferences of patients	Perceived inability to reconcile patient preferences with the use of SDM [27]
• Factors associated with shared decision-making as an innovation	
○ Lack of triability	Perception that SDM cannot be experimented with on a limited basis [30]
○ Lack of compatibility:	Perception that SDM is not consistent with one's own approach [30]
○ Complexity	Perception that SDM is difficult to understand and to put into use [30]
○ Lack of observability	Lack of visibility of the results of using SDM [30]
○ Not communicable	Perception that it is not possible to create and share information with one another in order to reach a mutual understanding of SDM [30]
○ Increased uncertainty	Perception that the use of SDM will increase uncertainty (for example, lack of predictability, of structure, of information [30]
○ Not modifiable/way of doing it	Lack of flexibility in the degree to which SDM is not changeable or modifiable by a user in the process of its adoption and implementation [30]
• Factors associated with environmental factors	
○ Time pressure	Insufficient time to put SDM into practice [30]
○ Lack of resources	Insufficient materials or staff to put SDM into practice [28]
○ Organizational constraints	Insufficient support from the organization
○ Lack of access to services	Inadequate access to actual or alternative health care services to put SDM into practice [28]
○ Lack of reimbursement	Insufficient reimbursement for putting SDM into practice [28]
○ Perceived increase in malpractice liability	Risk of legal actions is increased if SDM is put into practice [28]
○ Sharing responsibility with Patient*	Using SDM lowers the responsibility of the health professional because it is shared with patient

* Only for the facilitator assessment taxonomy

Both reviewers independently read each publication and identified the unit of text (a sentence or paragraph representing one idea) relevant to each of the main outcomes of interest (barriers or facilitators to the implementation of shared decision-making in clinical practice). Each unit of text was then coded according to the relevant and pre-established code list and entered into an Excel spreadsheet. Units of text which could not be coded were discussed by the two assessors and new codes were created as necessary, thus refining and expanding the preliminary list of codes. Discrepancies between the coders were resolved through iterative discussions. During the coding process, codes (e.g., lack of agreement with the applicability of shared decision-making to practice population based on the age of the patient) were aggregated into themes (e.g., lack of agreement with the applicability of shared decision-making to practice population based on the characteristics of the patient), which in turn were nested under the main theme – lack of agreement with the applicability of shared decision-making. Themes were ordered according to the number of studies in which they were identified.

### Quality assessment

Study characteristics were abstracted and included: country of origin, year and language of publication, main objective of the study, operationalization of shared decision-making, use of a conceptual framework to assess barriers and/or facilitators to the implementation of shared decision-making in practice, design of study within which barriers and facilitators were elicited, characteristics of participants, sampling strategy, response rate, and methodological approach, including data collection strategies.

Quality assessment of included studies was based on an existing framework and its set of validated tools[31,32]. This framework was selected because its authors provide reviewers with an extensive manual for quality scoring of quantitative, qualitative and mixed methods studies. The manual also includes definitions and detailed instructions[31]. Two reviewers (KG and FL) independently assessed the quality of each study. Discrepancies between the two coders were resolved through discussion. As the review did not involve human subjects, ethical approval for the study was not sought.

## Results

### Included studies

From PubMed, Embase, CINHAL, PsycINFO et Dissertation Abstracts, we screened 9580 references and assessed the full text of 170 documents. Thirty one publications[11,21,29,33-60] relating to 28 unique studies met our inclusion criteria, among which were two unpublished doctoral dissertations[33,42]. Three publications presenting additional but distinct data were from the same randomized controlled trial[21,35,36], and two were from the same cross-sectional study[54,55]. Thus, we abstracted data from each one of them. The number of publications/studies included at the various stages of the review process is shown in Additional file 2 (see Additional file 2).

### Study characteristics

The characteristics of included studies are shown in Additional file 3 (see Additional file 3). Studies were published in English, except for one that was published in French[53]. Most studies originated in the United Kingdom (n = 11)[21,35-39,43,45,47-49,56,58], followed by the United States (n = 8)[11,33,40-42,44,51,54,55], Canada (n = 4)[29,34,46,52], Netherlands (n = 2)[50,59], France (n = 1)[53], Mexico (n = 1)[57], and Australia (n = 1)[60]. One study from the Netherlands had enrolled health professionals from 11 countries (Austria, Belgium, Denmark, France, Germany, Israel, The Netherlands, Portugal, Slovenia, Switzerland, UK)[50]. Therefore, included studies reported data from health professionals in 15 countries. More than half of the studies were published in or after 2004 (n = 16)[34-36,43,49-60].

Only two studies were explicit in their use of a conceptual framework pertaining to the assessment of barriers and/or facilitators to the implementation of best practices in clinical practice[42,52]. Designs of study within which barriers and facilitators elicited included: cross sectional (n = 24)[11,29,33,34,37-46,48-51,53-55,58-60], randomized clinical trial (n = 3)[21,35,36,47,52], and before-and-after (n = 1)[57]. Ten studies were based on a probabilistic sampling frame[11,33,34,42,45,46,49-52]. Response rates were mentioned in 13 studies and varied from 42% to 97%[11,29,33,34,37,38,41,42,46,48,52,53,58]. Two studies did not report the number of participants[44,47]. In those that did, this number varied from 6 to 914. Overall, in studies that reported the number of participants, most of the participants were physicians (2481 out of a total of 2784 participants)[11,21,29,33-43,45,46,48-51,53-60]. Most studies used qualitative methods exclusively (n = 18)[29,37-39,41,43-45,47-51,54-56,58-60]. Six used quantitative methods exclusively[11,33,34,40,46,53], and four used mixed methods[21,35,36,42,52,57]. Data collection strategies included individual interviews (n = 15)[21,29,35,36,39,42,43,45,47,49-52,57-60], self-administered questionnaires (n = 10)[11,21,33-36,40,42,46,53,56], focus groups (n = 10)[21,35-38,43,44,48,52,54,55,57,60], and observation (n = 3)[41,47,57].

### Quality assessment of included studies

Table 2 shows the quality assessment of included studies. Except for two studies[44,56], most qualitative studies (n = 16/18) had an average score of 50% or more[29,37-39,41,43,45,47-51,54,55,58-60]. It is interesting to note that no qualitative study explicitly provided an account of reflexivity. In other words, researchers did not reflect on the influence that their backgrounds and interests might have had on their results. Overall, quantitative studies had an average score of 50% or more[11,33,34,40,46,53]. Mixed methods studies had an average score of 50% or more in both assessments (qualitative and quantitative)[21,35,36,42,52,57].

**Table 2:** Quality assessment of included studies

**Qualitative studies**																		
	**Study identification**																	
**Criteria**	**[60]**	**[37]**	**[38]**	**[39]**	**[29]**	**[41]**	**[43]**	**[44]**	**[45]**	**[51]**	**[54, 55]**	**[58]**	**[59]**	**[47]**	**[48]**	**[49]**	**[56]**	**[50]**
Question/objective sufficiently described?	2	2	2	2	2	1	2	0	2	2	2	2	1	2	2	2	2	2
Study design evident and appropriate?	2	2	2	2	2	2	2	0	2	2	2	1	1	1	1	2	1	2
Context for the study clear?	2	2	2	2	2	2	2	1	2	1	2	2	2	2	2	2	2	2
Connection to a theoretical framework/wider body of knowledge?	2	2	2	2	2	2	2	0	1	1	2	2	2	1	1	2	2	2
Sampling strategy described, relevant and justified?	1	1	1	1	1	2	1	0	2	2	1	1	1	1	1	2	1	2
Data collection methods clearly described and systematic?	2	2	2	2	2	2	2	0	2	2	2	2	2	2	2	2	1	2
Data analysis clearly described and systematic?	2	2	2	2	2	2	2	0	2	2	2	2	1	2	1	2	0	2
Use of verification procedure(s) to establish credibility?	0	2	2	0	1	0	1	0	1	0	0	0	0	0	0	0	0	0
Conclusions supported by the results?	2	2	2	2	2	2	2	2	2	2	1	2	2	2	2	2	0	2
Reflexivity accounted for?	0	0	0	0	0	0	0	0	0	0	0	0	0	0	0	0	0	0

**Total score/possible maximum score**	**15/20**	**17/20**	**17/20**	**15/20**	**16/20**	**15/20**	**16/20**	**3/20**	**16/20**	**14/20**	**14/20**	**14/20**	**12/20**	**13/20**	**12/20**	**16/20**	**9/20**	**16/20**

**Quantitative studies**																		
	**Study identification**																	
**Criteria**	**[53]**			**[33]**			**[34]**			**[40]**			**[11]**			**[46]**		

Question/objective sufficiently described?	2			2			2			2			2			2		
Study design evident and appropriate?	2			2			2			2			2			2		
Method of subject/comparison group selection or source of information/input variables described and appropriate?	1			2			2			1			2			2		
Subject (and comparison group, if applicable) characteristics sufficiently described?	2			2			2			2			2			2		
If interventional and random allocation was possible, was it described?	N/A			N/A			N/A			N/A			N/A			N/A		
If interventional and blinding of investigators was possible, was it reported?	N/A			N/A			N/A			N/A			N/A			N/A		
If interventional and blinding of subjects was possible, was it reported?	N/A			N/A			N/A			N/A			N/A			N/A		
Outcome and (if applicable) exposure measure(s) well-defined and robust for measurement/misclassification bias? Means of assessment reported?	2			2			2			2			2			2		
Sample size appropriate?	N/A			N/A			N/A			N/A			N/A			N/A		
Analytic methods described/justified and appropriate?	2			2			2			2			2			2		
Some estimate of variance is reported for the main results?	N/A			2			0			2			2			1		
Controlled for confounding?	N/A			N/A			N/A			N/A			N/A			N/A		
Results reported in sufficient detail?	2			2			2			2			2			2		
Conclusions supported by the results?	2			2			2			2			2			2		

**Total score/possible maximum score**	**15/16**			**18/18**			**16/18**			**17/18**			**18/18**			**17/18**		

**Mixed methods studies**																		
	**Study identification**																	
	**[21, 35, 36]**				**[42]**					**[57]**					**[52]**			

**Assessment of the qualitative component of the study**																		
**Criteria**																		
Question/objective sufficiently described?	2				2					2					2			
Study design evident and appropriate?	2				2					2					2			
Context for the study clear?	2				2					2					2			
Connection to a theoretical framework/wider body of knowledge?	2				2					2					2			
Sampling strategy described, relevant and justified?	1				1					1					1			
Data collection methods clearly described and systematic?	2				2					2					2			
Data analysis clearly described and systematic?	2				2					2					2			
Use of verification procedure(s) to establish credibility?	0				2					0					0			
Conclusions supported by the results?	2				2					2					2			
Reflexivity of the account?	0				2					0					0			
**Assessment of the quantitative component of the study**																		
Question/objective sufficiently described?	2				2					2					2			
Study design evident and appropriate?	2				2					2					2			
Method of subject/comparison group selection or source of information/input variables described and appropriate?	1				2					1					2			
Subject (and comparison group, if applicable) characteristics sufficiently described?	2				2					2					2			
If interventional and random allocation was possible, was it described?	2				N/A					N/A					N/A			
If interventional and blinding of investigators was possible, was it reported?	2				N/A					N/A					N/A			
If interventional and blinding of subjects was possible, was it reported?	2				N/A					N/A					N/A			
Outcome and (if applicable) exposure measure(s) well-defined and robust for measurement/misclassification bias? Means of assessment reported?	2				2					2					2			
Sample size appropriate?	2				N/A					2					N/A			
Analytic methods described/justified and appropriate?	2				2					1					N/A			
Some estimate of variance is reported for the main results?	2				2					1					N/A			
Controlled for confounding?	1				N/A					1					N/A			
Results reported in sufficient detail?	2				2					2					2			
Conclusions supported by the results?	2				2					2					2			

**Total score/possible maximum score**	**41/48**				**37/38**					**33/42**					**29/34**			

2: Yes

1: Partial

0: No

N/A: Not applicable

### Barriers and facilitators

Six publications focused solely on identifying barriers[21,40,45,48,53,59], while two focused solely on identifying facilitators[46,58]. Most focused on both barriers and facilitators[11,29,33-39,41-44,47,49-52,54-57]. Table 3 summarizes the barriers and facilitators that were reported. In order of frequency, the five most often identified barriers were: time constraints (18/28)[29,34-39,41-43,47,48,50,51,53-57,60], lack of applicability due to patient characteristics (12/28)[21,29,34,37,41,43,47-49,53-55,59], lack of applicability due to the clinical situation (12/28)[11,29,34,36-38,47-49,53-55,59], perceived patient preferences for a model of decision-making that did not fit a shared decision-making model (n = 9)[21,39,41,42,45,47,48,52,54,55], and not agreeing with asking patients about their preferred role in decision-making (n = 7)[11,38,40,42,43,50,59].

**Table 3:** Perceived barriers and facilitators to implementation of shared decision-making in clinical practice

**Factor as a barrier/facilitator**	**Barriers (number of studies in which this factor was identified as a barrier) [reference number]**	**Facilitators (number of studies in which this factor was identified as a facilitator) [reference number]**
**Knowledge**		
Lack of awareness/awareness	0	0
Lack of familiarity/familiarity	5 [29, 37, 39, 44, 49]	0
Forgetting	1 [41]	Not applicable
**Attitude**		
**Lack of agreement with specific components of shared decision-making/agreement with specific components of shared decision-making**		
• Interpretation of evidence	1 [29]	
• Lack of applicability/applicability		
○ Characteristics of the patient	12 [21, 29, 34, 37, 41, 43, 47-49, 53-55, 59]	4 [29, 35, 51, 54, 55]
○ Clinical situation	12 [11, 29, 34, 36-38, 47-49, 53-55, 59]	3 [37, 46, 51]
• Asking patient about his/her preferred role in decision-making	7 [11, 38, 40, 42, 43, 50, 59]	2 [42, 50]
• Asking patient about support or undue pressure	0	1 [34]
• Asking about values/clarifying values	0	0
• Not cost-beneficial/Cost-beneficial	3 [21, 29, 45]	1 [42]
• Lack of confidence in the developers/Confidence in the developers	0	1 [29]
**Lack of agreement in general/Agreement in general**		
• "Too cookbook" – too rigid to be applicable	2 [29, 48]	0
• Challenge to autonomy	1 [11]	0
• Biased synthesis	1 [29]	0
• Not practical/Practical	2 [29, 54, 55]	6 [29, 33, 41, 54-57]
• Total lack of agreement with using the model (not specified why)	2 [47, 50]	0
**Lack of expectancy/expectancy**		
• Patient's outcome	1 [33]	10 [33, 34, 37, 42, 46, 50-52, 54-56]
• Process expectancy	1 [56]	11 [11, 29, 33, 34, 36, 41, 42, 50, 51, 54, 55, 57]
• Feeling expectancy	0	1 [34]
Lack of self-efficacy/Self-efficacy	6 [21, 34, 37, 48, 50, 53]	0
Lack of motivation/Motivation	4 [21, 37, 51, 52]	15 [33, 35, 36, 38, 39, 41-44, 47, 49, 51, 52, 54, 55, 57, 58]
		
**Behaviour**		
External factors		
• Factors associated with patient		
○ Preferences of patients	9 [21, 39, 41, 42, 45, 47, 48, 52, 54, 55]	4 [34, 39, 42, 52]
• Factors associated with shared decision-making as an innovation		
○ Lack of triability/Triability	2 [29, 49]	1 [29]
○ Lack of compatibility/Compatibility:	2 [29, 33]	2 [29, 33]
○ Complexity/Ease of use	3 [21, 29, 45]	2 [29, 56]
○ Lack of observability/Observable	1 [29]	1 [29]
○ Not communicable/Communicable	3 [29, 38, 49]	0
○ Increase uncertainty/Decrease or manage one's own uncertainty	1 [45]	1 [37]
○ Not modifiable/Modifiable	1 [37]	1 [29]
• Factors associated with environmental factors		
○ Time pressure/Save time	18 [29, 34-39, 41-43, 47, 48, 50, 51, 53-57, 60]	3 [29, 42, 54, 55]
○ Lack of resources/Resources	4 [35, 47, 50, 53]	1 [50]
○ Organizational constraints/Organizational support	0	0
○ Lack of access to services/Access to services	2 [41, 60]	0
○ Lack of reimbursement/Reimbursement	0	0
○ Perceived increase in malpractice liability/Perceived decrease in malpractice liability	2 [47, 48]	0
○ Sharing responsibility with Patient	Not applicable	3 [37, 42, 51]

In order of frequency, the five most often identified facilitators were: motivation of health professionals (n = 15)[33,35,36,38,39,41-44,47,49,51,52,54,55,57,58], perception that shared decision-making will lead to a positive impact on the clinical process (n = 11)[11,29,33,34,36,41,42,50,51,54,55,57], perception that shared decision-making will lead to a positive impact on patient outcomes (n = 10)[33,34,37,42,46,50-52,54-56], perceptions that SDM is useful/practical (n = 6)[29,33,41,54-57], patient preferences for decision-making fitting a shared decision-making model (n = 4)[34,39,42,52], and characteristics of the patient (n = 4)[29,35,51,54,55]. Removing the two qualitative studies that had an average quality assessment score of less than 50% did not change these results.

Possible positive impacts on process included: believing that involving patients in decision-making promotes trust and honesty and, in turn, leads to better diagnosis and care[51]; helping patients address all their concerns[54]; improvement of doctor-patient relationship[50]; and providing health professionals with more background information about patients, which would enable them to judge patient needs and preferences better[50]. Possible positive impacts on outcomes included: patients' acceptance of advice and adherence to medication[50]; patients' satisfaction, either by reducing their worries or by increasing their understanding of disease and treatment options[50]; satisfaction with the decision made[46]; and better health outcomes[51].

## Discussion

In 1999, Frosch and Kaplan observed that there were few surveys of large samples of physicians on how they perceived shared decision-making[22]. Therefore, results of our systematic review are important because, to the best of our knowledge, they reflect the first to attempt to pull together the views of more than 2784 health professionals from 15 countries (most of them physicians) on barriers and facilitators to the implementation of shared decision-making in their clinical practice. These results should improve our understanding on how to effectively translate shared decision-making into health professionals' clinical practice.

Except for "lack of awareness," that is, the inability of health professionals to state that shared decision-making exists, the whole range of barriers initially proposed by Cabana and colleagues (1999) was identified[27]. Time constraint was the most often cited barrier for implementing shared decision-making in clinical practice. This is interesting because this was a major concern for health professionals across many different cultural and organizational contexts[29,34-39,41-43,47,48,50,51,53-57,60]. However, recent evidence about the time required to engage in a shared decision-making process in practice is conflicting[61,62]. Therefore, it will be important that future studies on the implementation of shared decision-making in practice investigate whether engaging in shared decision-making actually takes more time or not.

Lack of agreement with some specific aspects of SDM was the second and third most often cited theme of barriers for implementing shared decision-making in practice. It included the perceived lack of applicability due to the characteristics of patients and the lack of applicability due to the clinical situation. Perceived patient preferences for a decision-making model that does not fit SDM and not agreeing with asking patients about their preferred role in decision making were the fourth and fifth most reported barriers. Taken together, these are important because they suggest that health professionals might be screening *a priori*, which patients they believe are eligible for shared decision-making. This is of some concern because physicians may misjudge patients' desire for active involvement in decision making[63]. Therefore, in order to not increase inequity in health (patients who are not invited to be involved in decision making regarding their health, but who want to be), it will be important to address this barrier when implementing shared decision-making. We agree with Holmes-Rovner and her colleagues (2000) that interventions directed at patients and the system will be needed in order for shared decision-making to be implemented in actual practice[41].

The three most frequently reported facilitators clustered under attitude were: 1) motivation of health professionals to put shared decision-making into practice, 2) their perceptions of patient outcome expectancy (the perception that putting SDM into practice will lead to improved patient outcomes), and 3) process expectancy (the perception that putting SDM into practice will lead to improved health care processes). These results are congruent with the literature on the changing behaviour of health professionals[64,65]. Together, they suggest that anticipating positive outcomes before trying a shared decision-making approach may influence its implementation in practice. In other words, health professionals need to be able to perceive that the use of shared decision-making with their patients will have positive outcomes on the patients themselves or the processes of care. Although this might appear to be a logical approach when implementing shared decision-making in actual practice, how it will be achieved is still unclear.

Other interesting results from this systematic review are as follows. Lack of self-efficacy and lack of familiarity with SDM were mentioned as perceived barriers to the implementation of shared decision-making in six[21,34,37,48,50,53] and five studies[29,37,39,44,49], respectively. This suggests that strategies to implement SDM in clinical practice will need to include training activities targeting health professionals. Elwyn and colleagues (2004) have shown that it was possible to train physicians in shared decision-making[66]. However, future implementation studies in this field will need to focus on improving knowledge of how competencies in SDM can be sustained over time.

Notwithstanding its interesting results, our systematic review has some limitations. First, although we searched systematically and thoroughly for articles on perceived barriers and/or facilitators of implementing shared decision-making in clinical practice by health professionals, this is not a well-indexed field of research. Therefore, it is possible that some eligible studies were not included in this review. However, our search strategy had an estimated predictive positive value for key articles in shared decision-making of 10%–20%. Also, we were able to show that some of the barriers and facilitators were quite consistent across a large number of studies. Second, like other researchers [67-71], we believe that mixed methods systematic reviews (MMSR) constitute an emerging field of research that is still in need of tools to help reviewers synthesize results from qualitative, as well as from quantitative and mixed methods studies. In this review, as much as possible, we made our overall process explicit[72], including our quality assessment strategy. In a recently published MMSR on the impact of clinical information retrieval technology on physicians, Pluye and colleagues emphasized that "No one-size-fits-all tool exists to appraise the methodological quality of qualitative research"[67]. In our own review, we decided to use an existing set of tools[31,32] and provided a justification for our choice. In subsequent "sensitivity analyses," in which we ranked the studies from the lowest score to the highest score on the quality assessment score, we observed that in order to experience significant changes in the results, one would need to remove 11 and 8 studies with the lowest score for the assessment of barriers and facilitators, respectively. Third, we used an existing taxonomy to classify barriers and facilitators[27]. This taxonomy had been developed and used to abstract data from previous studies on barriers and facilitators to implementing clinical practice guidelines[27]. It also had been used in original data collection[28,73,74]. Other taxonomies have been proposed to perform original data collection in studies aimed at identifying implementation problems[75]. It is possible that the use of another taxonomy to content-analyse the data might have modified our results[28]. However, as mentioned by Espeland and colleagues (2003), the taxonomy that was used compares well with other such taxonomies[28]. Fourth, we did not contact the authors of the included studies to verify data interpretation[69]. However, the use of information from process evaluations and contact with authors does not appear to substantially change the results of systematic reviews of knowledge translation[76]. Lastly, quantification of themes was provided only "to gain an overview of the qualitative material," including the exploration of variation between studies[77].

## Conclusion

Given that implementation of shared decision-making in clinical practice is a relatively recent phenomenon of interest[23], we believe that the results of our systematic review have implications for the development of theory and for research in this field. The vast majority of the included studies did not report the explicit use of a barriers and/or facilitators assessment tool. In this systematic review, the explicit use of such a tool helped standardize the presentation of the many factors that are likely to influence the uptake of shared decision-making into clinical practice and facilitate the comparison between similar studies[78]. In turn, this should contribute to the elaboration of a theoretical base for translating shared decision-making into practice. As the fields of implementation science[79] and shared decision-making[80] mature, we hope that our understanding of factors that might hinder or facilitate the implementation of SDM into clinical practice will improve.

These results also can be used to help target priorities for future implementation studies of shared decision-making. For example, future studies on barriers and facilitators to the implementation of SDM could target nurses and pharmacists, two disciplines that have not been well studied but that have had a significant impact on the development of shared decision-making[6,41,81-87]. Overwhelmingly, published studies originated from the UK and the USA, suggesting clear leadership of their health service researchers in this area and possibly, larger contextual variables that will need to be taken into account in future studies. At the same time, this could be another limitation of our findings, as we need studies in all types of health care systems to fully understand cross-cultural and health care system impacts on the implementation of shared decision-making.

In this review, the same factor was sometimes identified as both a barrier and a facilitator to implementing shared decision-making. This situation has been reported previously in a study that explored the gap between knowledge and behaviour of physicians[88]. This points to the importance of developing a comprehensive understanding of the perceived barriers and facilitators. Therefore, a more in-depth exploration of these factors should be pursued in future qualitative studies. Quantitative studies also could be used to analyze surveys of large probabilistic samples of health professionals in this area. Items could be derived from the results of our systematic review. Multivariate statistical analyses could then be used to identify the barriers and facilitators that make the largest contribution to the outcome of interest: intention of health professionals to implement shared decision-making in their practice. Finally, these results provide some insight into the type of interventions that could be tested with more robust study designs in order to foster shared decision-making.

## Competing interests

All authors declare that they have no conflicting financial interests.

One of the authors of this review, IG, also is the author of one of the included studies.

## Authors' contributions

FL conceived the study, supervised KG's student project, validated the methods, validated the article selection, assessed the quality of the included studies, second-coded all included articles, analysed the results, and wrote the paper. KG selected the articles, assessed the quality of the included studies, first-coded all included articles, analysed the results, and reviewed the paper. IG validated the methods, analysed the results, and participated actively throughout the writing of the paper. FL is its guarantor.

## Supplementary Material

Additional file 1 DOC/Search strategies by data source Click here for file

Additional file 2 DOC/Number of publications/studies included at the various stages of the review process Click here for file

Additional file 3 DOC/Characteristic of included studies (n = 28) Click here for file
