# Supplementary material for: Barriers and facilitators to implementing shared decision-making in clinical practice: a systematic review of health professionals' perceptions
Source: Implement Sci. 2006 Aug 9;1:16. doi: 10.1186/1748-5908-1-16 (PMC1586024; doi:10.1186/1748-5908-1-16)
Supplement: Additional file 1 — DOC/Search strategies by data source [file 1748-5908-1-16-S1.doc]

Additional file 1. Search strategies by data source

| PubMed (PubMed) |
| --- |
|  |
| 1 "decision aids"[tiab] or "decision making"[tiab] or "decision making"[mh:noexp] or "decision support systems, clinical"[mh] or "decision support techniques"[mh:noexp] |
|  |
| 2 "patient involvement"[tiab] or "patient participation"[tiab] or "patient participation"[mh] |
|  |
| 3 "professional-family relations"[mh] or "professional-patient relations"[mh] or ((clinician*[ti] or doctor*[ti] or physician*[ti]) and (patient*[ti] or people*[ti])) |
|  |
| 4 "attitude of health personnel"[mh] |
|  |
| 5 "decision aids"[tiab] or "decision support techniques"[mh:noexp] |
|  |
| 6 "shared decision"[tiab] or "sharing decisions"[tiab] or "informed decision"[tiab] or "informed choice"[tiab] OR ((share*[ti] or sharing[ti] or informed[ti]) and decision*[ti]) |
|  |
| 7 (eng[la] or fre[la]) |
|  |
| ((2 AND (1 OR 3 OR 4)) OR (1 AND 3) OR (4 AND 5) OR 6) AND 7 |
|  |
| Embase (Embase.com) |
|  |
| 1 'decision aids' :ab,ti or 'decision making':ti,ab or 'decision support':ti,ab or 'decision making'/exp or 'medical decision making'/exp or 'decision support system'/exp |
|  |
| 2 (patient*:ti and (involv*:ti or participat*ti: or preference*:ti)) or 'involving *2 patients':ab or 'involving *2 patient':ab or 'involve *2 patients':ab or 'involve *2 patient':ab or 'patients *2 involvement':ab 'patient *2 involvement':ab or 'patients *2 involved':ab or 'patient *2 involved':ab or 'patients *2 participate' :ab or 'patient *2 participate' :ab or 'patients *2 participation' :ab or 'patient *2 participation' :ab or 'patient *2 preferences' :ab or 'patient participation'/exp |
|  |
| 3 ((physician*:ti or clinician*:ti) AND (family:ti or patient*:ti or parent*:ti)) or 'physicians *2 patients' :ab or 'parents *2 clinicians' :ab or 'clinicians *2 patients' :ab or 'doctor patient relation'/exp |
|  |
| 4 'physician attitude'/exp |
|  |
| 5 'decision aids' :ab,ti or 'decision support system'/exp |
|  |
| 6 'patient education'/exp or 'patient satisfaction'/exp |
|  |
| 7 'shared decision':ab,ti OR 'sharing decisions':ab,ti OR 'informed decision':ab,ti OR 'informed choice':ab,ti OR ((share*:ti or sharing:ti or informed:ti) and decision*:ti) |
|  |
| 8 [embase]/lim AND (french:la or English:la) |
| ((1 AND (2 OR 3)) OR (2 AND (3 OR 4)) OR (4 AND (5 OR 6)) OR 7) AND 8 |
|  |
| CINHAL (WebPSIRS) |
|  |
| 1((decision aids) or (decision making) or (decision support)) in ab,de,ti |
|  |
| 2((clinician* or nurse* or physician* or professional*) near2 (family or parent* or patient* or people*)) in ab,de,ti |
|  |
| 3((consumer* or patient*) near2 (involv* or participat* or preference*)) in ab,de,ti |
|  |
| 4((shared decision) OR (sharing decisions) OR (informed decision) OR (informed choice)) in ab,ti OR ((share* or sharing or informed) and decision*) in ti |
|  |
| 1 and (2 or 3) |
| 2 and 3 |
| 4 |
|  |
| PsycINFO (WebSPIRS) |
|  |
| 1((decision aids) or (decision making) or (decision support) or (group decision)) in ab,su,ti |
|  |
| 2((consumer* or patient* or client*) near2 (involv* or participat* or preference*)) in ab,su,ti |
|  |
| 3((clinician* or physician* or psychiatrist*) near2 (client* or family or parent* or patient* or people*)) in ab,ti,su or (therapeutic processes) in su |
|  |
| 4((shared decision) OR (sharing decisions) OR (informed decision) OR (informed choice)) in ab,su,ti OR ((share* or sharing or informed) and decision*) in ti |
|  |
| 5 la=french or la=english |
|  |
| ((1 AND (2 OR 3)) OR (2 AND 3) OR 4) AND 5 |

# 
