# Supplementary material for: Barriers and facilitators to implementing shared decision-making in clinical practice: a systematic review of health professionals' perceptions
Source: Implement Sci. 2006 Aug 9;1:16. doi: 10.1186/1748-5908-1-16 (PMC1586024; doi:10.1186/1748-5908-1-16)
Supplement: Additional file 2 — DOC/Number of publications/studies included at the various stages of the review process [file 1748-5908-1-16-S2.doc]

Additional file 2. Number of publications/studies included at the various stages of the review process

PubMed, Embase, CINHAL and PsycINFO

(1990-March 2006)

Publications potentially eligible

**(n = 9580)**

Publications not relevant

**(n = 9410)**

Publications potentially relevant and further assessed

**(n = 170)**

Publications not eligible

**(n = 139)**

- Not an original collection of data: 34
- Not about health professionals: 48
- Not about barriers or facilitators of shared decision-making*: 54
- Not in French or English: 1
- Duplicate: 2

Publications deemed eligible

**(n = 31)**

Eligible studies included in this review

**(n= 28)**

- 3 publications presenting additional but distinct data were from the same randomized controlled trial [21, 35, 36]
- 2 publications presenting additional but distinct data were from the same cross-sectional study [54, 55]
