# Supplementary material for: Barriers and facilitators to implementing shared decision-making in clinical practice: a systematic review of health professionals' perceptions
Source: Implement Sci. 2006 Aug 9;1:16. doi: 10.1186/1748-5908-1-16 (PMC1586024; doi:10.1186/1748-5908-1-16)
Supplement: Additional file 3 — DOC/Characteristic of included studies (n = 28) [file 1748-5908-1-16-S3.doc]

Additional file 3. Characteristic of included studies (n=28)

| **Country of origin, language,**  **year of publication** | **Principal objective of the study** | **Operationalization of shared decision-making** | **Conceptual framework for barriers/**  **facilitators assessment** | **Design of study within which barriers and facilitators elicited** | **Participants** | **Response rate** | **Methods** |
| --- | --- | --- | --- | --- | --- | --- | --- |
| Canada, English, 1997 [46] | To examine the variations in physicians’ opinions about the appropriateness and content of patient decision aids for women with node-negative breast cancer, and the criteria for evaluating the effectiveness of such aids. | Decision aids provide a structure for making a choice by systematically presenting information on treatment alternatives, risks, and benefits tailored to the patient’s particular clinical profile. | No | Cross-sectional | 144 oncologists | 87% | Quantitative.  Self-administered questionnaire. |
| United Kingdom, English, 1999 [37] | To explore the view of general practice registrars about involving patients in decisions and to assess the feasibility of using the shared decision-making model by means of simulated practice. | Informed choice is at the opposite end of the spectrum, where the patient is provided with “sufficient” information and the clinician withdraws from the decision process. Shared decision-making describes the middle ground. | No | Cross-sectional | 39 general practice registrars | 87 % | Qualitative.  Focus group. |
| United States, English, 1999 [40] | To explore the perceptions of health care providers regarding who is responsible for selected role functions in decision making. | Collaboration between nurses, physicians, and consumers in current health care delivery systems in decision making. | No | Cross-sectional | 5 administrators, 47 nurses, 11 physicians, 15 psychologists, social workers, 37 psychiatric technicians and 5 therapists | Not reported | Quantitative.  Self-administered questionnaire. |
| United States, English, 1999 [42] | To ascertain primary care and specialty physicians’ about more informed, actively involved patients as partners in health and medical care decisions – and the impact they believe consumers/patients being more informed and taking a more active partnership role in health and medical care decisions will have. | Demand management has been defined as the use of decision and self-management support systems to enable and encourage consumers to make appropriate use of medical care. | Yes [30, 89] | Cross-sectional | a) 914 physicians (379 in primary care and 535 specialists)  b) 13 primary care physicians, 7 specialty physicians, and 1 clinical psychologist | a) 42%  b) Not reported | Mixed methods.  a) Self-administered questionnaire.  b) Interviews. |
| United Kingdom, English, 2000 [38] | To explore and understand what constitutes the appropriate involvement of patients in decision making within consultation, to consider previous theory in this field, and to propose a set of competences and steps that would enable generalists to undertake shared decision-making in their clinical practice. | Involvement of patients in decisions. | No | Cross-sectional | 6 general practitioners | 80% | Qualitative.  Focus group. |
| United States, English, 2000 [41] | To determine the feasibility of shared decision-making programmes in fee-for-service hospital systems, including physicians’ office and in-patient facilities. | A model that brings patients into the decision-making process. It brings together consumer involvement in health care, evidence-based decision-making, and egalitarian models of the doctor-patient relationship. | No | Cross-sectional | 13 nurses, 7 social workers and administrators, and 14 physicians | 97% | Qualitative.  Observation. |
| United States, English, 2002 [44] | To enhance medical student learning about common clinical preventive services and to teach students how to inform and involve patients in shared decision-making about those services. | Patient involvement in shared decision-making. | No | Cross-sectional | Medical students | Not reported | Qualitative.  Focus group. |
| United States, English, 2002 [11] | To explore patients’ and physicians’ views of their roles in decision making and to determine perspectives of residents and patients on the amount of control each should have in health care decisions. | Decisions made jointly by the patient and the physician. | No | Cross-sectional | 45 residents in 7 residency programs | 63% | Quantitative  Self-administered questionnaire. |
| United Kingdom, English, 2002[47] | To examine the use of evidence- based leaflets on informed choice in maternity services. | Evidence-based leaflets to support consumer choice. | No | Randomized control trial | Health professional in 13 maternity units | Not reported | Qualitative.  Observation and interviews. |
| United States, English, 2003[33] | To elicit physicians’ opinions about the notion of a patient decision aid that could be used in shared decision making. | Process of interaction between patients who wish to be involved in making clinical decisions and their providers. It involves providing relevant clinical information, eliciting patient preferences, and arriving at a joint clinical decision that is consistent with the patient’s revealed personal values. | No | Cross-sectional | 248 endometriosis specialists and 112 generalists in gynaecology | 42% | Quantitative.  Self –administered questionnaire. |
| United Kingdom, English, 2003, 2004 and 2005 [21, 35, 36] | To explore, from paired doctor-patient interviews, participants’ perceptions of shared-decision making in the consultation and the level of consensus between the participants in the consultation process and to identify the experiences and views of professionals skilled in shared decision making and risk communication, exploring the opportunities and challenges for implementation. | A partnership between professionals and patients. | No | Randomized controlled trial | 20 general practitioners | Not reported | Mixed methods.  Questionnaires, interviews and focus group. |
| United Kingdom, English, 2003[39] | To identify the elements and skills required for a successful evidence-based patient choice consultation. | Evidence-based patient choice is defined as patient involvement in decision-making. | No | Cross-sectional | 11 general practitioners, 10 hospital consultants, 5 nurse practitioners, 11 academics, 8 lay people | Not reported | Qualitative.  Interviews. |
| Canada, English, 2003[29] | To investigate physicians’ perceptions of three patient decision aids and to identify factors perceived to encourage or discourage their possible uptake. | Decision aids are interventions designed to assist individuals confronted with therapeutic decisions; provide a structure for making a choice; present information on the options available, and the risks and benefits of theses options using probabilities tailored to the individual’s risk profile; describe what it would be like to live with the consequences of each choice; and may also clarify the personal importance or values individuals assign to the risks and benefits of therapy. | No | Cross-sectional | 20 family physicians, 12 gynaecologists, 16 respirologists and 19 medical specialists | 48% | Qualitative.  Interviews. |
| United Kingdom, English, 2003[45] | To explore the views of clinicians and lay people about the minimum benefit needed to justify drug treatment to prevent heart attacks, and to explore the rationale behind treatment decisions. | Most lay people want to make decisions for themselves, based on information provided by health professionals. | No | Cross-sectional | 4 general practitioners, 4 practice nurses and 18 lay people | Not reported | Qualitative  Interviews. |
| United Kingdom, English, 2003[48] | To explore the views of general practitioners of the practical application of shared decision making in their own and other participants’ real life practice. | Shared decision making implies that the doctor-patient relationship is moving towards a more active partnership with active involvement of patient. | No | Cross-sectional | 11 general practitioners | 55% | Qualitative.  Focus group. |
| Canada, English, 2004[34] | To explore the extent to which Ontario breast cancer specialists report practising shared decision-making with their patients, their comfort level with this approach, and perceived barriers and facilitators to implementation. | Shared decision-making implies the simultaneous participation of physicians and patients in all phases of the decision-making process. | No | Cross-sectional | 232 surgeons and 102 oncologists | Surgeons : 72%  Onco-logists : 79% | Quantitative.  Questionnaire. |
| United Kingdom, English, 2004[43] | To explore the way in which general practitioners in the UK manage the dual responsibilities of treating individual patients and making the most equitable use of National Health Service resources in the context of the policy of greater patient involvement in decision-making. | Private participation refers to the involvement of individuals in their own care and treatment while public participation refers to involvement in decision-making processes concerning service planning and delivery, service evaluations and consultations over future service provision. | No | Cross-sectional | 24 general practitioners | Not reported | Qualitative.  Focus group and interviews. |
| United Kingdom, English, 2004[49] | To explore whether newly qualified doctors feel adequately trained to discuss management with patients, their attitudes to the concept of sharing decisions about treatment with patients and their strategies for coping with managing patients. | Both patients and doctors disclose treatment preferences for a particular problem based on available evidence and personal preferences; negotiation then follows to build a consensus about the best treatment to follow. | No | Cross-sectional | 36 pre-registration house officers | Not reported | Qualitative.  Interviews. |
| Netherlands, English, 2004[50] | To determine specific barriers to the involvement of older patients in general practice care and to identify variations between countries. | Patient involvement is defined as enabling patients to take an active role in deciding about and planning their care. | No | Cross-sectional | 233 general practitioners in 11 European countries | Not reported | Qualitative.  Interviews. |
| United States, English, 2005 [51] | To identify and characterize physicians’ attitudes toward patient participation in decision-making and to gain insight into how they consequently think about and structure the decision-making process. | Involving patients actively in the clinical decision-making. | No | Cross-sectional | 53 academic and private practice physicians from primary care and surgical specialities | Not reported | Qualitative.  Interviews. |
| Canada, English, 2005[52] | To elicit the barriers and facilitators influencing the provision of decision support by call center nurses for callers facing values-sensitive health decisions and to explore the magnitude of these barriers and facilitators as perceived by the nurses. | Decision coaching is one-to-one guidance through a stepped process by someone who is supportive but neutral with respect to the decision. | Yes[90] | Randomized controlled trial | 108 registered nurses | Barriers survey:  52,8% | Mixed methods.  Interviews, focus groups, and self-administered questionnaires. |
| France, French, 2005[53] | To describe how paediatric residents involve children during medical decision-making and evaluate the relationship between practice patterns and residents’ characteristics. | To involve children during medical decision-making. | No | Cross-sectional | 45 paediatric residents | 75% | Quantitative.  Self-administered questionnaire. |
| United States, English, 2005 et 2006[54, 55] | To examine experiences of older persons and their clinicians with shared decision making and their willingness to use an SDM instrument. | Shared decision-making models are increasingly advocated to improve the process and outcomes of clinical decision making for patients with serious and chronic illnesses. | No | Cross-sectional | 5 nurses and 6 physicians | Not reported | Qualitative.  Focus groups. |
| United Kingdom, English, 2005[56] | To pilot test a decision aid for hypertension treatment based on decision analysis that incorporated guidance on the best options for patients, based on their personal preferences. | To provide individualised information to patients in a way that promotes their involvement in treatment decisions. | No | Cross-sectional | 2 consultant cardiologists, 2 general practitioners, 2 specialist nurses, 2 practice nurses | Not reported | Qualitative.  Self-administered questionnaire. |
| Mexico, English, 2005[57] | To report on a field test in Mexico that assessed the tool’s effectiveness in changing the counseling and decision-making process, and collected feedback from providers and clients. | A decision was considered shared when it emerge from the verbal exchange between the provider and the client, with each contributing. | No | Before-and-after | 9 doctors, 2 nurses and 2 social workers | Not reported | Mixed methods.  Interviews, observation and focus group. |
| United Kingdom, English, 2005[58] | To report the views of 21 general adult psychiatrists working in UK about their experiences of consultations involving discussion of antipsychotic medication. | Shared decision making involves choices that reflected patient’s wishes, negotiated agreements and a sense of partnership. | No | Cross-sectional | 21 consultant psychiatrists | 66% | Qualitative.  Interviews. |
| Netherlands, English, 2006[59] | To describe several barriers in shared decision making in an intercultural context. | Physicians are expected to inform their patients about all the possible benefits and risks of treatment options. In turn, patients are supposed to put forward their preferences, expectations, and concerns about the options suggested by the physician. | No | Cross-sectional | 18 physicians | Not reported | Qualitative.  Semi-structured interviews. |
| Australia, English, 2004[60] | To explore beliefs and expectations of general practitioners, consumers and pharmacists in relation to concordance to allow further exploration of the implementation of principles of concordance in Australia. | Concordance is an agreement or partnership between patient and prescriber about obtaining the best use of treatment, compatible with what a patient desires and is capable of achieving. | No | Cross-sectional | 9 pharmacists and 10 general practitioners | Not reported | Qualitative.  Focus group and semi-structured interviews. |
